# Justifications for Non-Consensual Medical Intervention: From Infectious Disease Control to Criminal Rehabilitation

**DOI:** 10.1080/0731129X.2016.1247519

**Published:** 2016-11-07

**Authors:** Jonathan Pugh, Thomas Douglas

**Keywords:** rehabilitation, punishment, informed consent, neuro-interventions, public health, coerced treatment, compulsion, infectious disease

## Abstract

A central tenet of medical ethics holds that it is permissible to perform a medical intervention on a competent individual only if that individual has given informed consent to the intervention. Yet it occasionally seems morally permissible to carry out non-consensual medical interventions on competent individuals for the purpose of infectious disease control (IDC). We describe two different moral frameworks that have been invoked in support of non-consensual IDC interventions and identify five desiderata that might be used to guide assessments of the moral permissibility of such interventions on either kind of fundamental justification. We then consider what these desiderata imply for the justifiability of carrying out non-consensual medical interventions that are designed to facilitate rehabilitation amongst serious criminal offenders. We argue that these desiderata suggest that a plausible case can be made in favor of such interventions.

A central tenet of medical ethics holds that it is permissible to perform a medical intervention on a competent individual only if that individual has given informed consent to the intervention. However, in some circumstances it is tempting to say that the moral reason to obtain informed consent prior to administering a medical intervention is outweighed. For example, if an individual’s refusal to undergo a medical intervention would lead to the transmission of a dangerous infectious disease to other members of the community, one might claim that it would be morally permissible to administer the intervention even in the absence of consent. Indeed, as we shall discuss below, there are a number of examples of public health authorities implementing compulsory or coercive measures for the purposes of infectious disease control (IDC).

The plausibility of the thought that non-consensual medical interventions might be justified when performed for the purpose of IDC raises the question of whether such interventions might permissibly be used to realize other public goods. In this article we focus on one possibility: whether it could be permissible to non-consensually impose certain interventions that alter brain states or processes through chemical or physical means on serious criminal offenders. We shall suggest that some such interventions might be permissible if they safely and effectively serve to facilitate the offender’s rehabilitation and thereby prevent criminal recidivism.^1^ We refer to brain interventions intended to serve these objectives as *neuro-correctives*.

Authorities in certain jurisdictions have already begun to explore the use of neuro-correctives. For instance, drug-addicted offenders are sometimes compelled to take medications that are intended to attenuate their addictive desires,^2^ and sex offenders in some jurisdictions may be compelled to take testosterone-lowering drugs as a part of their criminal sentence or as a condition of their parole.^3^ Moreover, as our understanding of the neural correlates of violent behavior increases, it seems plausible to speculate that we might develop effective neuro-correctives that involve the use of other pharmaceuticals^4^ or novel technologies such as deep brain stimulation,^5^ transcranial magnetic stimulation,^6^ and neuro-feedback.^7^

The ethical debate over the deployment of neuro-correctives has been increasing.^8^ Much of this debate has focused on their safety and effectiveness. We shall not examine these issues here. We simply assume, for the sake of argument, that the interventions we consider are effective and have negligible side effects. Other critics have questioned whether offenders could validly consent to undergoing such an intervention, if doing so were a condition of their early release from prison.^9^ In this article, we sidestep this contentious issue by assuming that the hypothetical recipients of neuro-correctives have *not* validly consented to undergoing the intervention. Instead, we shall argue that the considerations standardly offered in support of non-consensual interventions in IDC also offer considerable support to the permissibility of some safe and effective non-consensual neuro-correctives.^10^

First, however, some definitions. We shall adopt a definition of medical interventions that is perhaps broader than colloquial use, since we shall understand it to refer not only to typical medical procedures such as pharmacotherapies and surgical procedures—measures that involve some degree of bodily invasion—but also to measures that restrict an individual’s freedom of movement and association, insofar as these are used for medical reasons. For instance, we shall understand quarantine to qualify as a medical intervention. We will use the term *IDC interventions* to refer to medical interventions performed for the purposes of infectious disease control.

We shall use the term *non-consensual intervention* to refer to any intervention performed without the valid consent of the recipient of the intervention. It is possible to distinguish a number of different kinds of non-consensual intervention. For example, one might draw a distinction between compelled and coerced interventions. Following Feinberg, we may say that “an option is closed by compulsion when one alternative has been made impossible.”^11^ For instance, an individual may be compelled to undergo an intervention by being subjected to direct physical force. A compelled intervention is carried out without the recipient’s consent, and perhaps even despite his dissent. In contrast to compulsion, coercion does not make an alternative impossible, but rather destroys its appeal by increasing its cost.^12^ In contrast to the case of compulsion, in coercion, the individual does consent to the intervention, but the coercive pressure to which she has been subjected may *invalidate* that consent. We use the term *non-consensual intervention* to refer both to interventions performed without consent, and to interventions performed with invalid consent.

## I. Non-Consensual Interventions in IDC

The putative requirement to obtain valid consent before performing a medical intervention is standardly thought to derive from a reason to respect the prospective patient’s rights, which may include rights to personal autonomy and rights to freedom from bodily interference. Performing a medical intervention non-consensually is thought to be impermissible because it violates one or more of these rights.^13^ In the IDC cases of interest to us, this reason to respect the patient’s rights plausibly remains in place (it has not been forfeited). However, there is also a powerful reason to impose the medical intervention, since it will help to forestall the spread of an infectious disease. In a number of historical cases, this reason has been thought by many either to outweigh reasons to respect the individual’s rights, or to deprive these rights of their normal protective force, with the result that the intervention may permissibly be imposed non-consensually.

One type of medical intervention that has often been used for the purposes of IDC is vaccination. Vaccinations are amongst the most effective IDC interventions that societies can employ. For instance, broad vaccination coverage is largely responsible for the widespread immunity to measles and tetanus in modern society, and it led to the eradication of smallpox in the late twentieth century.^14^ Barring adverse side-effects, which are uncommon for most vaccines, and cases where an individual is insusceptible to the negative effects of an infectious disease, being vaccinated is typically beneficial to the vaccinated individual, since an effective vaccine will normally confer immunity to a particular infectious disease. However, effective individual immunization also confers benefits to third parties: immunizing a sufficient proportion of the population against a disease can disrupt its spread from person to person. In such a scenario of herd immunity, the fact that a sufficient majority of the population is immune to a particular disease confers a degree of protection even to those who have not been vaccinated.^15^

In light of this third-party benefit, some countries employ laws that could be described as introducing coercive pressure on individuals to undergo vaccination, insofar as they provide for the imposition of costs for vaccine refusal.^16^ For instance, in the majority of states in the USA, children must normally complete a vaccination schedule as a condition of entering public school.^17^ In some cases, it might be claimed that if the operative reason undergirding an individual’s choice to undergo the vaccination is that they desire to avoid these state-imposed costs of non-compliance (rather than, say, because they want the health benefits of being vaccinated), then that individual may qualify as being coerced in a manner that invalidates his consent.^18^

Vaccinations aim to prevent the spread of infectious disease by preventing individuals from becoming infected in the first place. However, states also impose other sorts of non-consensual medical intervention for IDC purposes. For example, in some jurisdictions it is legally permissible to compel the medical examination (and in rare cases even treatment) of persons suspected of carrying an infectious disease.^19^ The non-consensual examination and treatment of individuals carrying a dangerous infectious disease can aid IDC in two ways. First, effectively treating that individual will mean that that they will no longer be able to transmit the disease to others. Second, the treatment can help to restrict the development of drug-resistant pathogens, since one way in which a pathogen can develop drug resistance is by genetically evolving as a result of an incomplete treatment.^20^

Although exerting coercive pressure to undergo vaccinations and even compelling medical examinations and treatments is permitted in some jurisdictions, these are somewhat controversial non-consensual IDC interventions. The use of non-consensual quarantine and isolation, however, is often deemed to be less controversial (for reasons that we shall explore below). Both quarantine and isolation share the aim of separating certain individuals from the rest of the community in order to prevent or limit the transmission of infectious pathogens. The difference between the two lies in the diagnostic status of the detainee: in isolation, it is known that the detainee is infected, whereas quarantine involves the detention of individuals who have been (or are likely to have been) exposed to the infectious agent and are thus suspected of being (but not *known* to be) infected. Quarantine and isolation have long been used in response to outbreaks of infectious disease, particularly those for which no effective medical treatment is available.^21^ For instance, quarantine was widely used in response to the recent outbreak of Ebola in West Africa.

## II. Approaches To The Moral Justification of Non-Consensual Medical Interventions

In this section we shall delineate two standard moral justifications for non-consensual medical interventions in IDC: the constrained-consequentialist justification and the self-defense justification. We then identify some desiderata for assessing IDC interventions that are consistent with both justifications, before arguing in the subsequent section that these desiderata support the justifiability of at least some non-consensual neuro-correctives.

It might be argued that non-consensual interventions in IDC can be morally justified by appealing to classic utilitarian reasoning. On such a view, these interventions would be permissible if they are predicted to bring about at least as much aggregate well-being as any alternative course of action. However, few have been persuaded by such justifications. As T. M. Wilkinson points out, this sort of utilitarian thinking runs contrary to the belief, commonly held in biomedical ethics, that people have certain rights that “constrain the pursuit of the greater good.”^22^ Many theorists reject the simple utilitarian approach because of its failure to accommodate this belief amongst others; indeed, some may claim that this failure gives us reason to be skeptical of making any consequentialist considerations central to the justification of either medical or criminal-justice interventions, the two types of intervention with which we are concerned in this article. We shall now set out two alternative justifications to which defenders of non-consensual interventions have turned, which are both compatible with the belief that rights should constrain the pursuit of the good, but which may nonetheless be used to justify certain non-consensual interventions in IDC.

The first justification retains a broadly consequentialist approach but incorporates a richer account of the good than the utilitarian’s appeal to aggregate well-being, and sets certain side-constraints on the pursuit of this good. We shall call this version the *constrained consequentialist approach*. Lawrence Gostin offers an example of it in his influential *Public Health Law: Duty, Power and Restraint*. In introducing his account, Gostin writes: “The prime objective of public health law is to pursue the highest possible level of physical and mental health in the population, consistent with the values of social justice.” He later expands on this claim by suggesting that the twin aims that motivate public health interventions are “to advance human well-being by improving health, and to do so particularly by focusing on the needs of the most disadvantaged.”^23^

These passages suggest that Gostin deviates from utilitarianism in two respects. While he believes, like the utilitarian, that interventions must promote individual well-being, his appeals to social justice and the needs of the most disadvantaged suggest that, unlike the utilitarian, he does not regard aggregate well-being as the sole determinant of the good, but instead believes that the *distribution* of well-being across the community should be incorporated into the theory of the good. Moreover, the claim that the objective of public health law is to pursue high levels of health in a manner that is *consistent* with the values of social justice leaves open the possibility that the pursuit of good outcomes may be side-constrained—that is to say, there may be certain means to pursuing those outcomes that are ruled out, regardless of how good their effects would be. The nature of the constraints will depend on the theory of social justice that one adopts in cashing out the framework. Notably in claiming that public health ought to focus on the “needs of the most disadvantaged,”^24^ Gostin himself seems to implicitly endorse a fundamentally prioritarian account of social justice. Some prioritarians set side-constraints on the pursuit of the good by claiming that our pursuit of general well-being should be constrained by the principle that we must not select any option in which the worst-off individuals will be worse off than the worst-off individuals in any alternative distribution.^25^

A second approach to the justification of non-consensual IDC interventions treats the imposition of such interventions as an instance of justified self-defense. We may say that an agent is acting in self-defense if she is acting to prevent another (the attacker) from harming her.^26^ In the context of IDC, it has been suggested that it may be legitimate to think of an infected (or potentially infected) individual as an attacker, and other members of the public as potential victims who may be justified in defending themselves against the attacker, by, for example, imposing an IDC intervention.^27^

In what follows, we will not seek to adjudicate between constrained consequentialist and self-defense justifications for IDC interventions. Rather, we will deploy five desiderata that are commonly used to guide decisions regarding the deployment of non-consensual IDC interventions, and that are compatible with either kind of fundamental justification. We take these desiderata to collectively constitute the standard approach to decision-making regarding IDC interventions. We also take them to constitute *indicators* rather than *determinants* of the moral permissibility of public health interventions. These desiderata do not determine what is right and wrong in IDC; rather, they constitute a practically applicable set of heuristics that agents operationalising either of these fundamental justifications may apply in order to guide their assessments of permissibility. Furthermore, it should be noted that, though the desiderata we shall outline are consistent with both constrained consequentialist and self-defence justifications, their interpretation may depend on which of these fundamental justifications one accepts. As we wish to remain neutral between these fundamental justifications, we will, where necessary, leave open whether these desiderata should be interpreted in line with a constrained consequentialist or a self-defense approach.

## III. Five Desiderata To Guide The Moral Assessment of Non-Consensual Medical Interventions

The first desideratum is the gravity—by which we mean the moral weight—of the harm the intervention aims to prevent. This weight may depend, inter alia, on the magnitude of the harm, its qualitative importance, the likelihood or certainty with which it will occur, and its impact on distributive fairness.^28^ The graver the harm, the easier it will be to justify the intervention.

The second desideratum is the effectiveness of the intervention in preventing the harm. In IDC, this effectiveness will depend on the nature of the infectious disease, the nature of the intervention, and the context in which the intervention is to be deployed. Consider, for instance, the attempt to limit the spread of infectious disease by imposing mass quarantine: this solution will only be effective in preventing the spread of an infectious agent if it is possible to identify people likely to be incubating the infection, and if those people comply with the conditions of quarantine. Furthermore, the infectious agent must be transmissible in its pre-symptomatic or early symptomatic stages for quarantine to be effective in preventing further transmission.^29^

A third desideratum is that the interventions have low opportunity costs. The expenditure of economic resources on a public health intervention necessarily diverts funds from other potentially beneficial public projects, including projects that might themselves promote public health.^30^ The opportunity cost of an intervention is the value of the alternative use to which the resources invested in the intervention would otherwise have been put. The higher the opportunity cost, the more difficult it will be to justify the intervention. In many cases, determining the opportunity cost of an intervention is difficult because it is unclear how the resources would otherwise be spent. However, even in those cases it may be possible to make a (necessarily uncertain) estimate of the expected opportunity costs. Moreover, in other cases, opportunity costs are quite clear: consider the position of a committee that has been given a fixed budget to spend on fighting a new pandemic and is assessing competing proposals for the use of those funds.

The fourth and fifth desiderata are less straightforward, and so we will describe them in greater detail. The fourth desideratum calls for the IDC intervention to be the least restrictive of the available alternatives for preventing the harm. As the discussion in section I makes clear, many effective IDC interventions can impose significant burdens on the individuals they target. In overriding a competent individual’s refusal to consent, we arguably infringe his right to autonomy, and we may, depending on the intervention, also infringe other moral rights such as rights to bodily integrity, and freedom of movement and association. In view of these possible rights infringements, it has been suggested that public health authorities should realize their IDC goals through the use of the least restrictive alternative of the available options.^31^

The definition of restrictiveness here has important ramifications for the moral foundations of a least restrictive alternative desideratum. It might be claimed that the restrictiveness of an intervention depends on the extent to which it infringes upon the recipient’s moral rights.^32^ Notably, the understanding of rights that such an account might invoke need not assume that rights must trump all other considerations; an intervention may be permissible (on broadly consequentialist grounds or grounds of self-defense) even if it infringes another’s rights, as long as it is the intervention that infringes rights to the *least extent* of all available (effective) interventions. Of course, how the extent of rights infringements is to be determined is open to debate.

On this interpretation, the least restrictive alternative desideratum may be regarded as reflecting a deontological constraint on either the pursuit of the good (on constrained consequentialism), or self-protection (on a self-defense-based justification). In either case, it is an interpretation that is most naturally understood in deontological terms. However, some readers may be suspicious of this interpretation’s invocation of the language of rights. An alternative interpretation of the least restrictive alternative desideratum might instead claim that the restrictiveness of the intervention depends on the extent to which the intervention *harms* the subject; for instance, by frustrating certain interests they may have. On this reading, the least restrictive alternative desideratum may be understood to highlight the harms that non-consensual interventions can cause to the recipients of the intervention. On either a constrained consequentialist or self-defense justification, these harms inflicted by the intervention weigh against the harms that it averts (captured by the first desideratum) in determining its justifiability.

This brings us to the fifth desideratum, which is that the intervention must be proportionate to the threat the recipient of the intervention poses to others. This desideratum is most strongly associated with self-defense justifications, where the desideratum (understood as a guide to practical decision-making) can be seen as a direct operationalization of a proportionality requirement at the level of fundamental justification; self-defense accounts make the permissibility of protective measures dependent on the existence of proportionality. We will thus begin by briefly outlining how proportionality has been understood within self-defense justifications, before suggesting that a proportionality desideratum for non-consensual medical interventions can be accepted by proponents of a constrained consequentialist approach too, even though this approach includes no proportionality requirement at the level of fundamental justification.

Jeff McMahan succinctly captures the requirement of proportionality in self-defense in the following way: “The requirement of proportionality holds, roughly, that the harm inflicted in self-defence must not be excessive in relation to the threatened harm one seeks to avoid.”^33^ In order to establish whether a harm inflicted in self-defense is excessive, one must first assess the relative gravity of the harm inflicted by an act of self-defense, and the harm that the attacker can be expected to inflict in the absence of self-defense. On the *equivalent harm view* of proportionality, self-defense is proportionate only if the “harm that the force is intended to fend off is at least equivalent to the harm inflicted on the attacker.”^34^ On this view, inflicting fatal force in self-defense is only proportionate when doing so is intended to fend off a harm that is itself as grave as death.

However, other views take further factors into account. For instance, it might be argued that assessments of proportionality should acknowledge that the attacker may be liable to suffer defensive force by virtue of his culpability (or, on some views, his mere responsibility^35^) for the threat he poses, whilst the victim is not liable to suffer the threatened harm.^36^ On McMahan’s justice-based account, this asymmetry makes it permissible, as a matter of justice, to ensure that the threatening party, rather than the potential victim, suffers any harm necessitated by the threat posed.^37^ Moreover, on this view, the stringency of the proportionality restriction on the use of defensive force may vary in accordance with the threatening party’s degree of culpability (or responsibility) for the threat he poses.^38^ For our purposes here, the important implication of justice-based views of proportionality is that they allow for the possibility that it can be justifiable for an agent acting in self-defense to harm a morally responsible attacker *more* than the attacker would otherwise harm her.

In contrast to self-defense justifications, the idea of proportionality is arguably not fundamental to most constrained consequentialist justifications of IDC interventions. However, assessing whether an intervention can be justified on the constrained consequentialist approach requires assessing the net harmfulness of the intervention, where the most significant harms are likely to be harms to others that the intervention can be expected to prevent, and the harms that the intervention imposes on the recipient. Ceteris paribus, an intervention will be justified on this approach only if the expected harm to others that is averted is greater than the harm inflicted on the recipient, and this, on one interpretation (the equivalent harm view), is precisely what the proportionality desideratum requires. Satisfaction of the desideratum, thus interpreted, can therefore be regarded as an indicator of whether an IDC intervention can be justified on the constrained consequentialist approach.^39^

Certain constrained consequentialist approaches could also accommodate a proportionality desideratum that goes beyond the equivalent harm view and sanctions interventions that cause more harm than they prevent. For instance, a constrained consequentialist approach that incorporated desert into its account of the good could regard harms to culpable agents as less inimical to the good (because less underserved) than harms to innocent agents.^40^

It should be acknowledged that our use of the concept of proportionality here differs from the way in which proportionality is commonly invoked by retributivists in the criminal justice context. In this latter context, proportionality is commonly understood *retrospectively*: in order to ascertain whether a punishment is proportionate, we have to make a comparison between the harms that will be imposed by that punishment and the gravity of the wrong involved in the offender’s *past* action. In the case of retrospective proportionality assessments, we already know what crime the individual committed and have to judge what sort of punishment is proportionate to the harm imposed by that crime. In contrast, we are invoking proportionality in a *prospective* sense; in order to ascertain whether a preventative intervention that we are now going to impose is proportionate, we have to make a comparison between the harm that will be imposed by this intervention and the harm that individuals will otherwise bring about through their *future* action.^41^ This sense of proportionality arguably introduces epistemic difficulties: we may face barriers to knowing the extent of the harm that the agent will bring about, as well as her degree of culpability for it.

## IV. The Justification of Non-Consensual IDC Interventions and Neuro-correctives

Having outlined the five desiderata that we take to constitute the standard approach to the assessment of nonconsensual IDC interventions, we shall now consider what these desiderata might imply regarding the justification of non-consensual *neuro-correctives*, assuming that they are apt for the moral assessment of such interventions. Our discussion of non-consensual IDC interventions in the previous section suggests that there may be some circumstances in which it may be permissible to impose medical interventions non-consensually. For the sake of argument, we shall assume that non-consensual IDC interventions that are frequently employed in liberal democracies, or are regarded as serious contenders for such employment, are all permissible. We shall argue that considerations pertaining to the gravity of harm targeted, effectiveness, and opportunity costs count as strongly in favor of non-consensual neuro-correctives as they count in favor of these putatively permissible non-consensual IDC interventions, which include some forms of quarantine, coercive vaccination, and forced treatment. We shall then consider whether the least restrictive alternative and proportionality desiderata can be invoked to rule out the permissibility of neuro-correctives in a manner that does not also rule out the non-consensual IDC interventions. We shall argue that although some neuro-correctives would be more restrictive than other available methods for preventing recidivism, this is neither clearly the case for all neuro-correctives, nor clearly sufficient for establishing that these interventions would be impermissible. Similarly, we shall argue that many neuro-interventions would be proportionate (or not clearly disproportionate) to the harms they are intended to prevent.

In the preceding section, we outlined five desiderata that may be used to guide the moral assessment of non-consensual IDC interventions. One potential obstacle facing the translation of a moral framework from public health ethics to the context of criminal justice is that interventions in the latter context may have different aims. For instance, on retributive approaches to criminal justice, the purpose of criminal justice is to ensure that the offender gets his just deserts. Such approaches are backward-looking in the sense that they claim that the appropriateness of a particular correctional intervention depends ultimately on the offender’s past conduct (which renders him deserving of hard treatment), not on the effects of the intervention. As such, retributivism contrasts with forward-looking consequentialist approaches to criminal justice, which claim that the appropriateness of a particular correctional intervention is to be established by determining whether it will lead to good consequences. Strict retributivists are unlikely to be convinced that a public health moral framework is suitable for adoption within the criminal justice system, insofar as it does not incorporate backward-looking retributive elements that they deem to be central to criminal justice.

Naturally, we cannot settle debates about the justification and aims of criminal justice here. However, it should be acknowledged that rehabilitation has been understood to be a central goal of criminal justice on a wide range of penal theories,^42^ including both consequentialist theories and non-consequentialist moral education and paternalistic theories.^43^ We claim that insofar as rehabilitation is aptly construed as an appropriate goal of criminal justice (as it is on many theories), then this framework may be used to guide our moral assessments of how we may permissibly intervene in order to promote rehabilitation. This position is quite compatible with claiming that criminal justice ought also to incorporate other retributive elements; those who make this claim might feel it necessary to impose other forms of intervention on offenders in order to meet these aims, and to supplement the framework we present here with backward-looking considerations. The relative weights of the desiderata we present here and other retributive desiderata (such as retrospective proportionality) will depend on the comparative weight that one affords to different aims of criminal justice.

Furthermore, the desiderata that we have presented may be justified by either a constrained consequentialist approach or a deontologically grounded theory of self-defense. Notably, the latter can be understood as partially integrating both retributive and crime-prevention elements, since on this approach force may only permissibly be used on aggressors, even if the primary purpose is to avert harm.^44^ That said, the limit of using force only on aggressors does not ensure compliance with negative retributivism, since some aggressors may arguably be innocent or only very mildly culpable. Moreover, even when the desiderata are understood as being undergirded by a constrained consequentialist theory, the approach is not vulnerable to some of the criticisms of advocating rehabilitation as a central unconstrained consequentialist aim of punishment. For instance, one such criticism is that an unconstrained consequentialist approach would seem to allow for *any* sort of harsh treatment that would achieve the goal of rehabilitation, including interventions that would traditionally be ruled out as disproportionate by retributive approaches. However, the constrained consequentialist approach we have outlined can avoid this criticism by incorporating a proportionality requirement that limits the sorts of interventions we may impose on offenders in the name of rehabilitation.^45^

Let us turn, then, to applying the five desiderata outlined in the previous section to the case of neuro-correctives. Consider first the gravity of the harm that neuro-correctives seek to prevent. It seems plausible to claim that neuro-correctives could be used to prevent serious harms, since many crimes cause serious harm (both physical and mental) to their victims. Indeed, in some cases (most obviously in the case of murder), the harm caused to the victim may be comparable in seriousness to the harm of his contracting a lethal infectious disease. Furthermore, criminal offending leads to other indirect costs for other members of society, since resources must be spent in order to, inter alia, provide support to victims of crime and apprehend and punish criminal offenders; in the UK, the cost to the taxpayer of reoffending is estimated to be £9.5 billion to £13 billion per year.^46^

However, even if criminal offending often causes harm to its victims, it might be argued that there are nonetheless significant differences between the harms caused by criminal offending and the harms caused by the spread of infectious disease. First, it might be claimed that the certainty with which the harm will occur in the absence of intervention is typically higher in IDC than it is in criminal justice: we cannot predict whether a criminal offender will reoffend with the same degree of accuracy with which we can predict that an individual carrying an infectious disease will transmit it to others. Although various risk-assessment instruments have been used to assist sentencing and release decisions in both the U.S. and the U.K.,^47^ they all have a number of well-documented problems.^48^ Recidivism can thus currently be predicted only with limited accuracy, and false positive assessments are likely in this context.^49^

However, this does not mean that the harms associated with criminal recidivism can be predicted with less confidence than those associated with infectious disease; public health authorities sometimes have to use risk-prediction instruments that have comparably limited predictive accuracy. Consider cases in which the transmission of an infectious agent depends on the vector engaging in certain behaviors, such as in sexually transmitted infections. In assessing whether an infected individual poses a threat to public health, authorities must determine the risk of that agent’s engaging in unprotected sexual intercourse. It seems doubtful that they could make such an assessment with a significantly greater degree of certainty than an assessment of whether a violent criminal offender is likely to reoffend. Another case in which public health authorities may face significant uncertainty in their predictions of threats to public health is the implementation of quarantine. In quarantine, healthy individuals can be subjected to compulsory detention just because they have been exposed to an infectious agent, even though they themselves may not be infected, and the threat they actually pose to others is thus uncertain.

Nonetheless, as we described above, individuals carrying sexually transmitted infections have been subjected to non-consensual treatment in the name of public health in certain liberal democracies, and quarantine is widely practiced across such jurisdictions. For that reason, and on the assumption that these interventions are morally permissible, the mere fact that the prediction of criminal recidivism might be highly uncertain is not *in itself* sufficient for establishing that the IDC moral framework cannot appropriately be applied in an assessment of compulsory neuro-correctives, as the prediction of infectious disease risks may also be highly uncertain.

Alternatively, it might be argued that the *aggregate* magnitude of the harm that could be prevented through the use of non-consensual neuro-correctives is minimal in comparison to that which could be prevented through the use of non-consensual IDC interventions. The latter often aim to prevent the spread of serious infectious diseases that might otherwise have the capacity to spread throughout a large population. In contrast, whilst criminal reoffending might entail certain indirect economic costs, it seems that an individual offender is likely to cause serious direct individual harm to only a small number of people compared to the large numbers of people who would be caused such harms by the spread of an infectious disease.

The strength of this argument turns on two empirical claims: first, that the scope of the harm caused by individual offenders is likely to be small, and second, the claim that permissible non-consensual IDC interventions prevent harms with large scope. However, it is not clear that either claim is universally true. First, it seems possible that a criminal offender could be likely to directly harm a large number of people; for instance, if the offender has previously been convicted of terrorist offences. Second, non-consensual IDC interventions, including some that are regarded as serious contenders for use in liberal democracies, are not always intended to prevent serious direct harm to large numbers of people; they may be imposed in response to infectious diseases that are typically non-lethal. For example, it has been argued that influenza vaccinations should be mandatory for all health care workers, even though some have suggested that comparatively few deaths are attributable to the forms of influenza against which such vaccinations protect.^50^ As such, the potential scope of the harms that neuro-correctives might prevent may not differ significantly from those that might be prevented by putatively permissible IDC interventions.

Consider now the second and third desiderata: effectiveness and opportunity cost. It is reasonable to believe that some neuro-correctives will score at least as well on these desiderata as putatively permissible IDC interventions. Admittedly, since many of the neuro-correctives discussed above are only in developmental stages, there is no robust data on whether these interventions would be effective in preventing recidivism. However, the limited data on the use of chemical castration to prevent recidivism in sex offenders might leave us with cause for optimism in this regard. In an extensive meta-analysis, Friedrich Lösel and Martin Schmucker found an 11.1% recidivism rate amongst sex offenders who had been castrated following the offense compared to a 17.5% recidivism rate amongst non-treated offenders.^51^ Second, there seems no reason to suppose that the opportunity costs associated with neuro-correctives would be any greater than those associated with IDC interventions. In fact, neuro-correctives would be very similar in kind to the sorts of pharmacological interventions that might be used in IDC, and thus likely to involve similar resource expenditure.

Accordingly, it seems that some neuro-correctives would score at least as well as putatively permissible IDC interventions on the desiderata of gravity of harm averted, effectiveness and opportunity cost. This suggests that, if these IDC interventions are indeed permissible, then non-consensual neuro-correctives are not universally ruled out as impermissible by these three desiderata. Nevertheless, it might be argued that the least restrictive alternative and the proportionality desiderata rule out the use of neuro-correctives. We shall conclude by considering each of these desiderata in turn.

First, however, it is necessary to explore further the nature and implications of the least restrictive alternative desideratum. Suppose that we adopt the rights-based understanding of the desideratum explored in the previous section. An assessment of an intervention’s restrictiveness will then take into account the nature of the rights that the intervention violates, the frequency and duration of the violation, and the number of people whose rights are violated.^52^ Some comparisons of restrictiveness will be relatively straightforward; for instance, two interventions might differ only on one of the above dimensions. To illustrate, compare the use of directly observed therapy (DOT), where the subject is required to take the prescribed dosage of his medication under observation, to compelled treatment in IDC.^53^ Whilst DOT arguably infringes on the individual’s rights in important ways, it seems clear that DOT is less restrictive than compelled treatment within a hospital setting, holding fixed the number of doses and people affected, since it does not infringe upon the individual’s right to freedom of movement and association to the same extent, insofar as it does not involve the imposition of involuntary confinement.

Comparisons of the restrictiveness of different interventions become less straightforward when they differ on more than one dimension; for example, where one intervention violates right M but not right N, and the other violates right N but not right M. Similarly, comparisons become less straightforward when one intervention violates the rights of more people, and the other violates the rights more extensively.

Nevertheless, in some cases it may be possible to make a somewhat credible comparison of the restrictiveness of the available alternative interventions. In the context of IDC, if our aim is to prevent further transmission of an infectious pathogen, we might compare compelled treatment with DOT, for example, and it might seem clear that DOT is less restrictive. In the context of using neuro-correctives in criminal justice, in order to determine whether or not such interventions represent the least restrictive means to achieving the end of preventing recidivism, we must compare them with the other means available to us. It might be argued that a comparison of neuro-correctives with alternative means of preventing criminal recidivism will reveal neuro-correctives to be *more* restrictive than some available alternatives. Let us assume that we are comparing (i) subjecting one individual to a non-consensual neurocorrective, and (ii) subjecting the same individual to incarceration of the least restrictive kind necessary to retain whatever anti-recidivist effect incarceration has.^54^ (We shall also assume the rights-based understanding of the least restrictive alternative desideratum, although our conclusions would also hold on the harm-based understanding.)

The right to freedom of movement and association is clearly relevant to this comparison. Incarceration seems to infringe on an offender’s right to freedom of movement and association, assuming that there is such a right and that it is not waived by the commission of a criminal offence. Moreover, infringements of this right may need to be continued for many years if the anti-recidivist effect is to be maintained. Neuro-correctives, by contrast, plausibly involve only a lesser violation of this right. Even if the offender were required to present at a specified location for repeated administrations of a neurocorrective, this requirement would clearly be a less extensive infringement of this right than incarceration.

Nonetheless, it seems that there is a strong case for claiming that the use of non-consensual neuro-correctives would be more restrictive than other penal methods, because such interventions would violate other rights that might be deemed more important than the right to freedom of movement and association. First, insofar as neuro-correctives would entail some degree of physical invasion, non-consensual neuro-correctives would violate the offender’s right to bodily integrity in a manner that is comparable to some of the non-consensual medical interventions surveyed above – for example, vaccinations – as well as compulsory medical examination and treatment. Although many jurisdictions permit non-consensual quarantine and isolation, which both seriously infringe upon an individual’s rights to freedom of movement and association in the name of public health, such jurisdictions often prohibit non-consensual medical treatment, which would plausibly involve a serious violation of a right to bodily integrity (although it is not clear how much more serious this violation is than that involved in the non-consensual medical examinations). A plausible explanation for the acceptance of this position is that the right to bodily integrity is seen as more important than the right to freedom of movement and association, ceteris paribus.

However, it is not obvious that this is so, given the great importance of freedom of movement and association to maintaining our most valuable personal relationships.^55^ Moreover, even if the right to bodily integrity *is* more important, other things being equal, this difference in importance may be offset by a difference in the *extent* of the rights infringements in the cases of interest to us. Incarceration involves very extensive intrusions on freedom of movement and association; neuro-correctives, by contrast, need not always involve extensive intrusions on bodily integrity (for example, of the sort involved in surgical procedures) but might rather involve relatively moderate forms of physical interference, such as, for example, the administration of a drug via nasal spray, or the administration of a very weak electric current (as in transcranial direct current stimulation). One of us has argued elsewhere that, taking into account both the importance of the rights and the extent of their infringement, the rights infringements involved in incarceration are at least as grave as those involved in the administration of a drug via injection,^56^ which would plausibly make them more grave than those involved in the administration of a drug in less physically invasive ways.

There is thus room to question whether all neuro-correctives are more restrictive than incarceration by virtue of their physical invasiveness alone. However, opponents of neuro-correctives might argue that there is another way non-consensual neuro-correctives would be more restrictive than incarceration by appealing to what we might term the offender’s right to mental integrity, or his right to have “the freedom to think one’s own thoughts and to have one’s own personality.”^57^ Whilst incarceration can undoubtedly bring about some mental effects, an important moral difference between incarceration and neuro-correctives seems to be that during the course of incarceration these effects are mediated by psychological processes.^58^ In contrast, neuro-correctives would bring about profound mental effects directly through the biological modulation of the brain states on which mental states supervene. Furthermore, these effects may also be unintended when they are brought about during the course of currently employed penal methods, while it is the express purpose of neuro-correctives to bring them about. In view of the fact that neuro-correctives involve intentionally changing another person’s mental states, those scholars who stress the importance of relational conditions of autonomy might claim that the use of neuro-correctives involves the exertion of third-party *control* over another person’s mental states in a way that non-intentional effects on a prisoner’s mental states do not.^59^ It thus seems plausible to claim that the former are more mentally invasive and arguably more autonomy-undermining than traditional penal methods.

Perhaps it is plausible to maintain that neuro-correctives are more restrictive than incarceration by virtue of the mental interference they involve, or by virtue of the combination of physical and mental interference. Again, however, it may be possible to respond by conceding that rights to mental integrity and bodily integrity are more important than rights to freedom of movement and association, while maintaining that the extensiveness of the violation of the latter right involved in incarceration exceeds the extensiveness of the violation of the former rights involved in the administration of some neuro-correctives. After all, just as some neuro-correctives might be administered through means that involve only moderate forms of physical invasion, so too might some neuro-correctives involve only mild or moderate forms of mental invasion: consider a neurocorrective that has a very local effect on one behavioral disposition (say, towards impulsive violence) that is not central to the offender’s self-conception or personality.

Perhaps more importantly though, the use of non-consensual medical interventions in IDC suggests that the fact that an intervention is more restrictive than an alternative is not alone sufficient to establish that carrying out the intervention would be impermissible (this is the reason why we referred to the least restrictive alternative desideratum as a *desideratum*, not a requirement). One of the main problems with assessing the moral justification of different sorts of non-consensual interventions in IDC or criminal justice are the trade-offs that will often occur between the desiderata we delineated in the previous section. In particular, in many cases there will likely be a trade-off between the effectiveness of an intervention and its restrictiveness. For instance, in the context of IDC, although DOT is less restrictive than compelled treatment, it also increases the subjects’ opportunity to avoid taking their medication. In such cases, we must judge that the least restrictive alternative is going to be sufficiently effective in achieving its aim to warrant our employing that intervention instead of a more restrictive alternative that is likely to be more effective.

The above considerations present a problem for opponents of neuro-correctives, since it is widely agreed that incarceration is ineffective at preventing recidivism amongst criminal offenders.^60^ A similar charge can be made against psychosocial rehabilitation. Consider, for example, the use of such programs for sexual offenders. Despite the wide use of these programs amongst this population of offenders, a number of critics have argued that there is simply no good evidence that such programs are effective in preventing reoffending.^61^ With this in mind, even if a non-consensual neurocorrective is not the least restrictive means of preventing recidivism available, the use of such an intervention may nonetheless be morally permissible according to the moral framework outlined above, if this intervention is substantially more *effective* at preventing recidivism than other less restrictive means of achieving that end.

One way in which an opponent of neuro-correctives might respond to this point is to appeal to the proportionality desideratum. It might be argued that interventions with a degree of restrictiveness that is above a proportionality threshold would be ruled impermissible, even if they are the least restrictive of the available alternatives, or offer the best combination of restrictiveness and effectiveness. It could then be argued that neuro-correctives would, by virtue of their physical and mental invasiveness, invariably fall above this threshold. Moreover, it might be argued that many IDC interventions are dis-analogous with neuro-correctives on this front: since many IDC interventions benefit their victim in some sense (in so far as they might treat individuals or prevent them from suffering an infectious disease), they are generally less restrictive, all things considered, than neuro-correctives, and are more likely to fall below a proportionality threshold of the sort that we are discussing here.^62^

At this juncture, it seems that the precise interpretation of the proportionality desideratum becomes particularly salient. Consider first the equivalent harm view of proportionality. On this view, the use of a non-consensual neurocorrective will only be proportionate if it prevents harms that are comparable to the harm caused by the intervention. As we have explained above, it seems that the use of some (although not all) non-consensual neuro-correctives might involve profound harms. It might be claimed that this feature restricts the scope of morally permissible non-consensual neuro-correctives to scenarios in which they are expected to prevent a similar degree of harm.

However, as we have seen, other interpretations of the proportionality desideratum allow that it can be permissible to impose harms on a threatening party that are greater than the harm they themselves pose. On these accounts, if a criminal offender is believed to *culpably* pose a significant risk to the life of just one other person, then that may be enough to justify imposing significantly harmful preventative interventions on him. Crucially for our purposes, unlike many recipients of IDC interventions, it seems that the recipients of neuro-correctives can plausibly be understood to *culpably* pose a threat in a way that may plausibly justify imposing a harm on them that is greater than the harm that they themselves threaten to bring about. Moreover, as we have seen, significantly restrictive interventions have often been thought permissible in the context of IDC. These factors together cast significant doubt on the suggestion that neuro-correctives would invariably lie above the proportionality threshold.

## V. Conclusion

Drawing on two theoretical frameworks, we have delineated five desiderata that are often thought apt to guide moral assessments of non-consensual medical interventions in the context of IDC. We then employed these desiderata to assess the moral justifiability of non-consensual medical interventions performed for the purposes of criminal rehabilitation. We argued that at least some neuro-correctives could be highly effective at preventing considerable harms with few opportunity costs, thus satisfying three of the desiderata to a high degree. After making that argument, we pointed out that even though neuro-correctives need not always involve profound harms to the recipient of the sort that are described in the literature, they may not be the least restrictive alternative rehabilitative measure available. However, in assessing the non-consensual use of such interventions, considerations of restrictiveness must be weighed against the desideratum of effectiveness; even if less restrictive rehabilitative alternatives are available, we may nonetheless have sufficient reason to use neuro-correctives if they are more effective in preventing recidivism. Finally, we suggested that the fact that many serious criminal offenders are culpable for the threat of future harm they pose suggests that significantly restrictive preventative interventions may satisfy the proportionality desideratum on plausible justice-based accounts of proportionality.

One concern that might be raised in response to our arguments is that they might plausibly be extended to justify the use of non-consensual crime-preventing medical interventions on individuals who have not previously committed a criminal offence, but who are still predicted to pose a threat of significant harm. This might be deemed problematic in our current political and legal culture, in which we are reluctant to punish people for their states of character, and instead believe that sanction should only be imposed for past conduct. Although we cannot offer a full treatment of this issue here, it should be noted that our arguments do not extend straightforwardly to this practice. Currently, one significant barrier to reliably predicting the risk of offending amongst non-offenders is that one of the main statistical predictors of future criminal offending is past criminal offending; for obvious reasons, this predictive factor would be absent in the risk assessment of non-offenders. Given the potentiality for error and abuse, and the various reasons we have to try to minimize the extent to which the state may legitimately exercise coercive power over citizens, this epistemological barrier represents a sufficient reason to only permit the use of neuro-interventions as a post-conviction sanction.

However, we suggest that this barrier is epistemological rather than moral. Suppose, completely fantastically, that we could, with 100% accuracy and reliability, predict that an individual with no history of criminal offending will culpably bring about a significant harm, and that psychosocial rehabilitation would not be effective in preventing him from doing so. What should we do in the absence of the epistemological barrier? Whilst we should be wary of state abuse of this power, in the theoretically pure case of a thought experiment, we suggest that the “bullet” of believing that it could be morally permissible to carry out a non-consensual neurocorrective on such an individual to prevent this harm becomes easier to bite; it is difficult to see how the mere fact that this person’s criminal offence lies in the future rather than the past should be an impediment to preventing the harm that he will otherwise certainly bring about.

## Notes

1 Notice that it might be possible to prevent recidivism in ways that do not involve the rehabilitation of the offender. For instance, incapacitating offenders by incarcerating them can be understood as an anti-recidivist measure. Since it seems that neuro-interventions that serve to incapacitate an offender would face additional moral problems in comparison to rehabilitative neuro-correctives, we shall consider in this article only those interventions that might be used to prevent recidivism by facilitating rehabilitation.

2 See Eley et al., “Drug Treatment and Testing Orders”; Hough et al., “Impact of Drug Treatment.”

3 See Thibaut et al., “WFSBP Guidelines.”

4 See Crockett et al., “Serotonin Modulates Behavioral Reactions”; Crockett et al., “Serotonin Selectively Influences Moral Judgment”; Crockett et al., “Serotonin Modulates Striatal Responses.”

5 See Lu, Wang, and Kosten, “Stereotactic Neurosurgical Treatment.”

6 See Young et al., “Disruption of Right Temporoparietal Junction.”

7 See Sitaram, Caria, and Birbaumer, “Hemodynamic Brain-Computer Interfaces.”

8 See T. Douglas, “Criminal Rehabilitation”; Shaw, “Direct Brain Interventions”; Ryberg, “Punishment.”

9 See Bomann-Larsen, “Voluntary Rehabilitation?” For relevant arguments that are specific to the context of chemical castration, see Rosati, “Study of Internal Punishment”; McMillan, “Kindest Cut?”; Vanderzyl, “Castration as an Alternative.”

10 Derk Pereboom has discussed the possibility of providing a non-retributive justification of incarceration that is compatible with free-will skepticism by appealing to similarities between the justification of quarantine and incarcerating criminal offenders on the assumption that they do not deserve to be punished. See Pereboom, *Free Will, Agency, and Meaning*; Pereboom,“Free Will Skepticism”; and Pereboom, *Living without Free Will*. More recently, Gregg Caruso, in “Free Will Skepticism,” has developed Pereboom’s model within a broader justificatory framework of public health ethics. We are sympathetic to many of the claims made in these works, particularly Caruso’s claim that we should give greater priority to the prevention of recidivism in criminal justice. However, unlike Pereboom and Caruso, our interest in this article is not to establish a non-retributive justification of incarceration but rather to investigate the extent to which the moral justification of non-consensual medical interventions in public health might be used to justify the use of comparable non-consensual interventions in criminal justice. Furthermore, we consider alternative accounts of the justification of non-consensual public health interventions to Caruso’s. In fact, the arguments we make here may be understood to extend Caruso’s conclusions regarding the non-retributive justification of incarceration to the use of neuro-correctives as anti-recidivist measures.

11 Feinberg, *Moral Limits of Criminal Law*, vol. 3, 190.

12 See ibid., 191.

13 See Beauchamp and Childress, *Principles of Biomedical Ethics*; Faden and Beauchamp, *History and Theory*; Crisp, “Medical Negligence”; Meisel, Roth, and Lidz, “Toward a Model.”

14 See Ehreth, “Global Value of Vaccination”; Gostin, *Public Health Law*, 376.

15 See Anderson and May, “Immunisation and Herd Immunity”; Anderson and May, “Vaccination and Herd Immunity.”

16 In contrast to attaching costs to vaccine refusal, Australia offers financial incentives to citizens to get vaccinated. See Australian Government, “Immunisation Requirements.” For comprehensive reviews of vaccination policies, see Salmon et al., “Compulsory Vaccination”; El Almin et al., “Ethical Issues.”

17 See Center for Disease Control, “SchoolVaxView.”

18 See Nozick, “Coercion”; Feinberg, *Moral Limits of Criminal Law*, vol. 3, 190; Zimmerman, “Coercive Wage Offers”; Beauchamp and Childress, *Principles of Biomedical Ethics*.

19 Reynolds v. McNichols, 488 (10th Cir. 1973). State of Delaware, “Title 16,” 524–5; UK Health and Social Care Act 2008.

20 See Gostin, *Public Health Law*, 415; Iseman, “Treatment of Multidrug-Resistant Tuberculosis.”

21 See Gensini, “Concept of Quarantine in History.”

22 Wilkinson, “Contagious Disease and Self-Defence,” 343.

23 Gostin, *Public Health Law*, 21.

24 Ibid.

25 Other prioritarians claim only that situation of the worst-off should be given greater weight than the that of others in ranking alternative possible distributions.

26 See Doggett, “Recent Work.”

27 Wilkinson, “Contagious Disease and Self-Defence.”

28 There is, of course, much disagreement about the requirements of distributive fairness, but, on many accounts, harms to worse-off individuals detract more from distributive fairness than harms to better-off individuals.

29 Schabas, “Severe Acute Respiratory Syndrome.” Note that the marginal effectiveness should be considered; that is, the effectiveness the intervention will add to other means of preventing harm that will be pursued regardless. Suppose, for example, that either a medical intervention or an education program would alone lower the risk of harm from infectious disease compared to a baseline in which neither intervention were pursued. Suppose by contrast that the education program will be pursued regardless, and that the medical intervention adds nothing to the effectiveness of the education program—that is, that the risk of harm is not reduced by adding the medical intervention to the education program. In this context, the effectiveness of the medical intervention should be regarded as zero.

30 See Gostin, *Public Health Law*, 64–8.

31 See ibid., 68. Relatedly, the literature on self-defense frequently invokes a condition of minimal force; permissible acts of self-defense must involve the use of only the *minimum force* that is necessary to avert the threat. This application can be regarded as one gloss on the least restrictive alternative desideratum. See Wilkinson, “Contagious Disease and Self-Defence.”

32 See Gostin, *Public Health Law*, 68.

33 McMahan, *Ethics of Killing*, 412.

34 Uniacke, “Proportionality and Self-Defense,” 258. Although Uniacke herself argues against the equivalent harm view, she cites both Rodin, *War and Self-Defense*, 48–9, and Leverick, *Killing in Self-Defence*, 153, as advocating it.

35 McMahan suggests that moral responsibility and culpability can be distinguished in the following way: an agent’s degree of culpability is a function of a number of variables, including, inter alia, whether the agent poses the wrongful threat intentionally, recklessly, or negligently; whether the agent has an excuse and how strong that excuse is; and the magnitude of the threatened harm. However, even if the agent is fully excused for posing a threat of harm, he may still be liable to defensive harm if he bears some degree of *moral responsibility* for posing a threat of harm. For instance, McMahan suggests that agents who pose a threat of harm because they are acting under an irresistible influence may still be morally responsible for their act even if they are not culpable. This moral responsibility is sufficient to render the agent liable to some degree of defensive harm, on his view, although the proportionality restriction on that defensive harm will be more stringent by virtue of the fact that the agent is not culpable. See McMahan, *Killing in War*, 159–73.

36 See Uniacke, “Proportionality and Self-Defense,” 261.

37 See McMahan, “Self-Defense and Culpability,” 766; McMahan, *Ethics of Killing*, 401–11; McMahan, *Killing in War*, chap. 4.

38 See McMahan, *Ethics of Killing*, 412; McMahan, *Killing in War*, chap.4; McMahan, “Proportionate Defense.”

39 It is merely an indicator, rather than a determinant, because the intervention may also cause or prevent other harms that would need to be included in the consequentialist calculus, and because deontological side constraints may rule the intervention out even if it satisfies this desideratum.

40 Indeed, a consequentialist theory of this kind could hold that harms to culpable agents contribute positively to the overall good. See, generally, Miller, *Social Justice* for an influential desert-based theory of social justice. See Rawls, *Theory of Justice* for criticisms of desert-based theories.

41 McMahan draws a similar distinction in his discussion of the difference between proportionality in punishment and self-defense. See McMahan, “Proportionate Defense,” 22–3.

42 See Bentham, *Theory of Legislation*; Cullen, *Reaffirming Rehabilitation*; Carlen, “Crime, Inequality and Sentencing.”

43 See Hampton, “Moral Education Theory of Punishment”; Morris, “Paternalistic Theory of Punishment.”

44 See Golash, *Case against Punishment*, chap. 5.

45 Notice that even if one holds that criminal justice should not at all aim at rehabilitation, our argument will still have interesting implications. That public health ethics justifications for non-consensual interventions seem also to work for neuro-correctives suggests that there is a prima facie case for using neuro-correctives. Thus, even if one thinks that the latter could not permissibly be incorporated within criminal justice systems, our arguments suggest that there may be reasons to use neuro-correctives *outside* criminal justice; for instance, as part of some kind of separate public protection system.

46 See U.K. Ministry of Justice and Home Office, “2010 to 2015 Government Policy.”

47 See K. S. Douglas et al., “Assessing Violence Risk”; Fazel et al., “Use of Risk Assessment Instruments”; Harrison, “Dangerous Offenders.”

48 See K. S. Douglas et al., “Assessing Violence Risk” for a discussion.

49 A further higher-order problem with actuarial instruments is the number of difficulties in measuring their predictive accuracy. See Rice and Harris, “Violent Recidivism.”

50 See Offley, “Influenza Vaccination”; Doshi, “Influenza.”

51 Lösel and Schmucker, “Effectiveness of Treatment.” It should be acknowledged that these numbers refer to a sample that includes offenders who have received either chemical or physical castration.

52 See Gostin, *Public Health Law*, 68.

53 DOT has commonly been used for patients suffering from tuberculosis, and it is also occasionally used in the treatment of HIV. See Bayer and Wilkinson, “Directly Observed Therapy”; Mitty et al., “Directly Observed Therapy.”

54 It might be argued that we cannot legitimately appeal to a comparison between incarceration and neuro-correctives. See Barn, “Can Medical Interventions Serve?” For example, it might be argued that the two interventions are intended to serve different aims. On many accounts, the justification for incarcerating criminal offenders often appeals to retributive or deterrent purposes, rather than the prevention of individual recidivism. However, as we claimed above, the prevention of recidivism plausibly plays some role in the justification of incarceration. This is most clearly so in cases of civil commitment. In such cases, offenders have already served a sentence that has been deemed sufficient to serve the goals of deterrence and retributivism, but they nonetheless remain incarcerated on the basis that criminal justice authorities believe that there is a significant risk that they would reoffend if released. Note also that the least restrictive kind of incarceration compatible with retaining an anti-recidivist effect would plausibly be far less restrictive than prevailing kinds of incarceration, which arguably involve harms and rights violations that are gratuitous from the point of view of preventing recidivism, and indeed may serve to encourage it. It might be objected at this point that taking incarceration as the relevant comparator is illicit, since criminal justice systems frequently have at their disposal less restrictive means of preventing recidivism, such as psychosocial rehabilitation programs. We will respond to this objection in our discussion below by appealing to the importance of the effectiveness desideratum and its relation to the least restrictive alternative desideratum.

55 See T. Douglas, “Criminal Rehabilitation.”

56 Ibid.

57 Farah, “Emerging Ethical Issues,” 1126.

58 See Bublitz and Merkel, “Crimes Against Minds.”

59 See Taylor, *Practical Autonomy and Bioethics*, chap. 1.

60 See Cullen, Jonson, and Nagin, “Prisons Do Not Reduce Recidivism”; Chen and Shapiro, “Harsher Prison Conditions”; Smith, Goggin, and Gendreau, “Effects of Prison Sentences.”

61 See Ho and Ross, “Cognitive Behaviour Therapy”; Dennis et al., “Psychological Interventions for Adults.”

62 However, there are other contexts in which interventions that seem comparably restrictive to neuro-correctives are used non-consensually for the prevention of similarly problematic harms. For instance, many jurisdictions have mental health legislation that allows competent but mentally disordered individuals to be treated non-consensually with mind-altering drugs when they constitute a threat to themselves or others.
